# Index event of cerebral amyloid angiopathy (CAA) determines long-term prognosis and recurrent events (retrospective analysis and clinical follow-up)

**DOI:** 10.1186/s42466-021-00152-x

**Published:** 2021-09-27

**Authors:** Andrea Wagner, Christiane Groetsch, Sibylle Wilfling, Karl-Michael Schebesch, Mustafa Kilic, Marjan Nenkov, Christina Wendl, Ralf A. Linker, Felix Schlachetzki

**Affiliations:** 1 Department of Neurology, University of Regensburg, Bezirksklinikum, Universitätsstraße 84, 93053 Regensburg, Germany; 2 Department of Neurosurgery, University Hospital Regensburg, Franz-Josef-Strauss-Allee 11, 93053 Regensburg, Germany; 3 Institute for Neuroradiology, University Hospital Regensburg and Bezirksklinikum, Universitätsstraße 84, 93053 Regensburg, Germany

**Keywords:** Cerebral amyloid angiopathy, Intracerebral hemorrhage, Acute ischemic stroke, Cerebral microbleedings, Cortical superficial siderosis- long term outcome

## Abstract

**Background:**

The modified Boston criteria (mBC) define the probability for the diagnosis of cerebral amyloid angiopathy (CAA). Its initial clinical presentation differs from asymptomatic cerebral microbleedings (cMBs), acute ischemic stroke (AIS), cortical hemosiderosis (cSS), to lobar ICH (lICH).

**Methods:**

Retrospective analyses and clinical follow-ups of individuals with at least mBC “possible” CAA from 2005 to 2018.

**Results:**

149 patients were classified in subgroups due to the index event: lICH (n = 91), AIS (n = 32), > 3 cMBs only (n = 16) and cSS (n = 10). Patients in the lICH subgroup had a significantly higher percentage of single new lICHs compared to other groups, whereas patients in the AIS-group had a significantly higher percentage of multiple new AIS. cMBs as index event predisposed for AIS during follow up (*p* < 0.0016). Patients of the cMBs- or cSS-group showed significantly more TFNEs (transient focal-neurological episodes) and lower numbers of asymptomatic patients (for epilepsy and TFNEs) at the index event than patients with lICH or AIS (*p* < 0.0013). At long-term follow-up, the cMBs- and cSS-group were characterized by more TFNEs and fewer asymptomatic patients.

**Conclusions:**

A new classification system of CAA should add subgroups according to the initial clinical presentation to the mBCs allowing individual prognosis, acute treatment and secondary prophylaxis.

**Supplementary Information:**

The online version contains supplementary material available at 10.1186/s42466-021-00152-x.

## Background

CAA is responsible for up to 20% of non-traumatic lICH surpassed only by hypertension [1–5]. Non-specific secondary preventative measures against recurrent CAA-related lICH include strict antihypertensive management and avoidance of additional cerebrovascular risk factors [1, 4, 6–8].

Next to lICH and due to accumulation of β amyloid in (sub)cortical and leptomeningeal arterioles, and capillaries leading to fragile vessels, CAA results in other phenotypes such as acute cortical subarachnoidal hemorrhages (cSAH) and cSS, asymptomatic cortical cerebral cMBs and AIS and white matter lesions [1, 2, 4]. Due to higher utilization of cerebral magnetic resonance imaging (cMRI) with iron-sensitive sequences also in elderly patients, CAA-diagnosis becomes more frequent. However, in patients with an indication for oral anticoagulation, this circumstance poses a therapeutic conflict. The existing data on the long-term prognosis of patients according to the number of cMBs do not differentiate between hypertensive and CAA-related cMBs, and data on CAA-patients with cSS. Thus, there is an unmet need for a widespread tool to determine CAA prognosis.

## Methods

We screened our data files between 2005 and 2018 for patients with the diagnosis of at least possible CAA due to the mBC [9]. The patients were grouped into four groups according to the index event eventually leading to the CAA diagnosis: lICH, AIS, cMBs, and cortical SAH/cSS (cSS-group). Classification of CAA in subtypes was performed according to the primary manifestation of CAA in the individual patient (= index event): patients with a (symptomatic) lICH were grouped to the lICH group, incidental findings on MRI consistent with CAA during work-up for other brain diseases (number of cMBs ≧ 5) were grouped to the cMB group (reasons for cMRI: dementia in 4 patients, grand mal seizures in 5 patients, focal epileptic seizures in 2 patients, and TIA/TFNE in 7 patients), patients with symptomatic or asymptomatic cSS or cSAH after exclusion of other reasons were grouped to the cSS/cSAH group and patients who presented with an AIS detected by cMRI fulfilling the diagnostic criteria for CAA were grouped to the AIS group. According to the modified Boston criteria, patients in the lICH-group could solely be included by cCT findings. Patients of the other three groups were included on basis of cMRI.

The following information was extracted from medical records for the index event and all patient contacts with our neurological ward between 2005 and 2018: age and gender, further events, treatment with antiplatelet and anticoagulation agents, TFNEs and epileptic seizures. In March 2018, we performed long-term follow up of all patients—either by clinical examination or by paper-bond or telephone interview of the patients or their relatives or primary caretakers. The simplified modified Rankin Scale (smRS) was calculated for all patients with sufficient available data [10, 11]. For the other patients, we tried to at least get information whether or not the patient is still alive. Patients with only the information that they were alive at the last follow-up without knowledge of the mRS, are represented by the “alive 0–5” group. All available MRI data were examined for cMBs using the MARS criteria and for cSAH and cSS [12, 13]. cSS was further sub-grouped in focal (less than 4 affected sulci) and disseminated cSS (more than 4 affected sulci) [14]. Additionally, we searched for recent ischemic lesions and white matter hyperintensities (classified using the Fazekas score) [15, 16]. In patients with more than one MRI, the progress/regress of the number of cMBs and the intensity of cSS was monitored as well as the appearance of new AIS.

The age distribution between the subgroups was initially analyzed with ANOVA and, after reaching statistical significance here (*p* < 0.05), each subgroup was compared to the other three subgroups using two-sided t-tests (level of significance, adjusted to multiple testing: *p* < 0.0125). Additionally, the age at the index event was analyzed depending on gender. For this, again a two-sided t-test with a level of significance of 0.05 was applied. The distribution of sex between the groups was evaluated by a chi-square test on a 2 × 4 contingency table (sex vs. subgroups). Values of mRS in long term follow-up were compared by a chi-square test on the contingency table (mRS vs. index event). Long-term follow-up regarding TFNEs, seizures, and asymptomatic patients was evaluated based on binomial testing (H0 being the mean in group 1, H1 being group 2 > group 1 (TFNE, seizures) resp. group2 < group1 (asymptomatic). In case a patient presented both epileptic seizures as well as TFNEs we chose the seizures subgroup for statistical analysis. P-values were considered significant if < 0.008 (adjusted for multiple testing). The number of cMBs was evaluated with ANOVA (significant *p*-values < 0.05) and Pearson’s correlation coefficients were calculated to evaluate the correlation between age and number of cMBs. cSS in the lICH group was compared to cSS in the other pooled groups by a two-tailed t-test again (level of significance = 0.05). The assessment of recurrent events in long-term follow-up was performed employing binomial testing with the given alternative hypotheses (greater than the other three subgroups each). Adjusted for multiple testing, p-values smaller than 0.016 were considered significant. The numbers of AIS in follow-up MRI for the different subgroups were compared using chi-square with a significance level of 0.05 again. Additionally, the number of AIS in follow-up MRI of the lICH-group was compared to the AIS-group with binomial testing (significance level 0.05).

## Results

### Classification of the patient collective

We identified 154 patients meeting the criteria for at least possible CAA (42 possible CAA, 112 probable CAA). Of these, 5 patients had to be excluded due to missing informed consent. Of the remaining 149 patients, 91 patients (61%) were patients of lICH-group, followed by patients with an AIS (21.5%), only cMBs (10.7%) and cSS (6.7%). The average age of the patients was 73.42 years (range 55–98 years, standard deviation = 8.11 years) with significant differences between the 4 subgroups (ANOVA: *p* < 0.015). Patients in the AIS-group had an average age of 69.50 years and were significantly younger than the rest of the collective (*p* < 0.0018, two-tailed t-test). 60.4% of the patients were male with no significant differences regarding subgroups (chi-square: *p* > 0.74).

Women were significantly older at index event than men (*p* < 0.01, mean age (women)/(men) = 74.23/ 70.65 years, (Additional file 1; Table 1).

**Table 1:** Basical clinical patient information for the four subgroups

	ICH	AIS	cMBs	cSS
Number of patients	91	32	16	10
Average age	74	70	73	76
Men (%)	57.1	65.6	68.8	60.0
Single platelet aggregation (%)	26.4	25.0	31.1	60.0
Phenprocoumon (%)	6.6	6.3	0.0	10.0
nOAK (%)	3.3	0.0	6.3	0.0
Other anticoagulation/antithrombotic therapy (%)	3.3	6.2	0.0	0.0
Unknown anticoagulation/antithrombotic therapy (%)	4.4	3.1	0.0	0.0
No anticoagulation/antithrombotic therapy (%)	56.0	59.4	62.5	30.0

### Long-term patient outcome

44% of patients were in our neurological ward between their index event and follow-up in 2018. We were able to perform any long-term follow-up in 89% of our collective; in 69% we obtained detailed information. The mean follow-up time was 6.9 years. Follow-up information included presence of TFNEs and epileptic seizures, recurrent events such as AIS or lICH and long-term mS outcome.

**Fig. 1:**
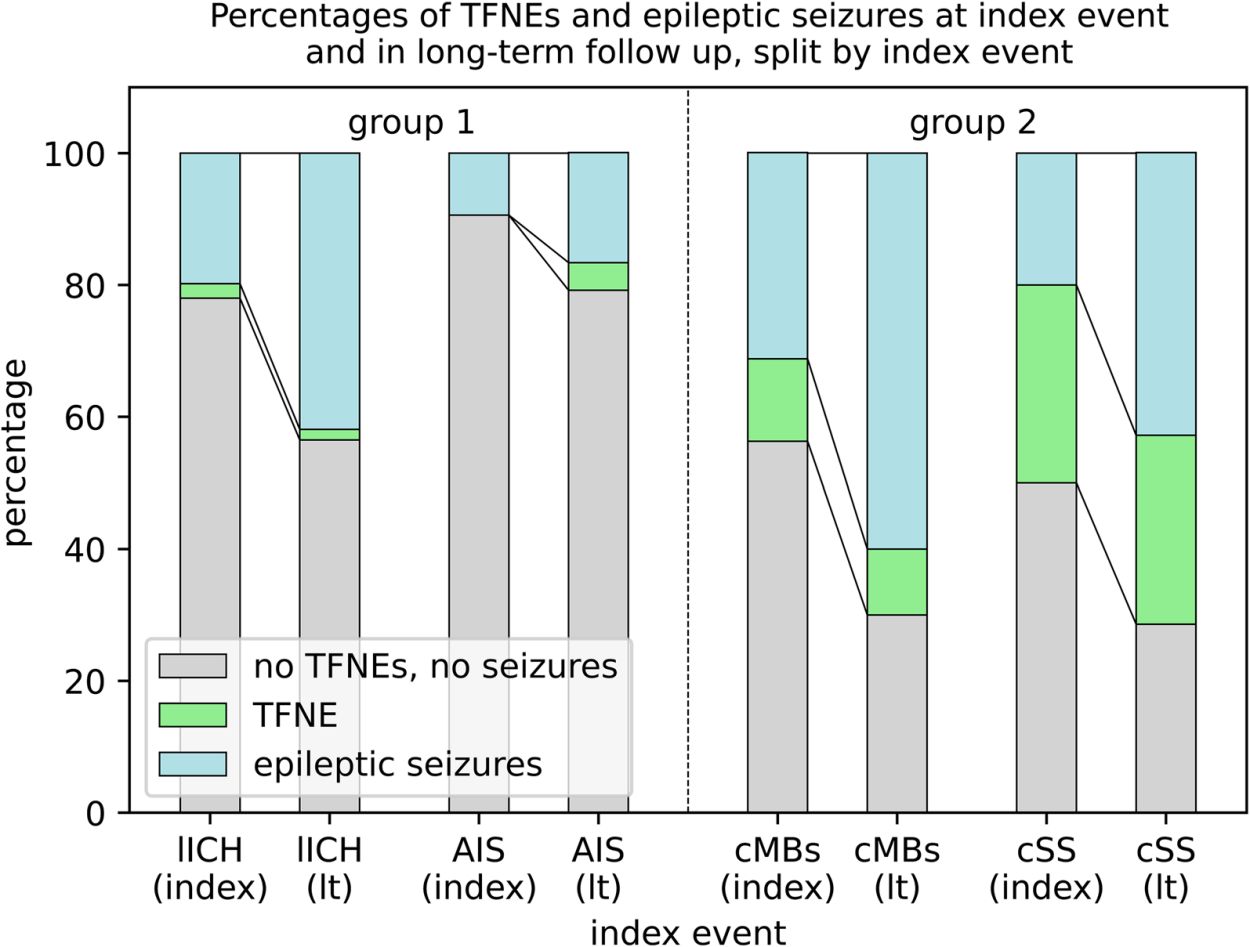
TFNEs and epileptic seizures for each subgroup at index event and upon long term follow up. In all subgroups, the percentage of epileptic seizures increased over time while the percentage of TFNEs stayed constant. (index = index event; lt = longterm follow-up)

Regarding transient events, patients of the cMBs- or the cSS-group showed significantly more TFNEs and lower percentages of asymptomatic patients at the index event than patients with lICH or AIS (*p* < 0.0013). At long-term follow-up, this effect was still present (*p* < 0.0067) (Fig. 1; Additional file 1: Appendix 2).

With regard to new structural brain damage, 48% of patients with long-term follow-up suffered from one recurrent lICH or AIS. The highest percentage of recurrent events was in the AIS- and cMBs-group (both 57.1%). Patients with AIS presented evenly with recurrent single AIS or lICH with 14.3% (2 patients) in each group. One patient suffered recurrent both, AIS and lICH.

**Fig. 2:**
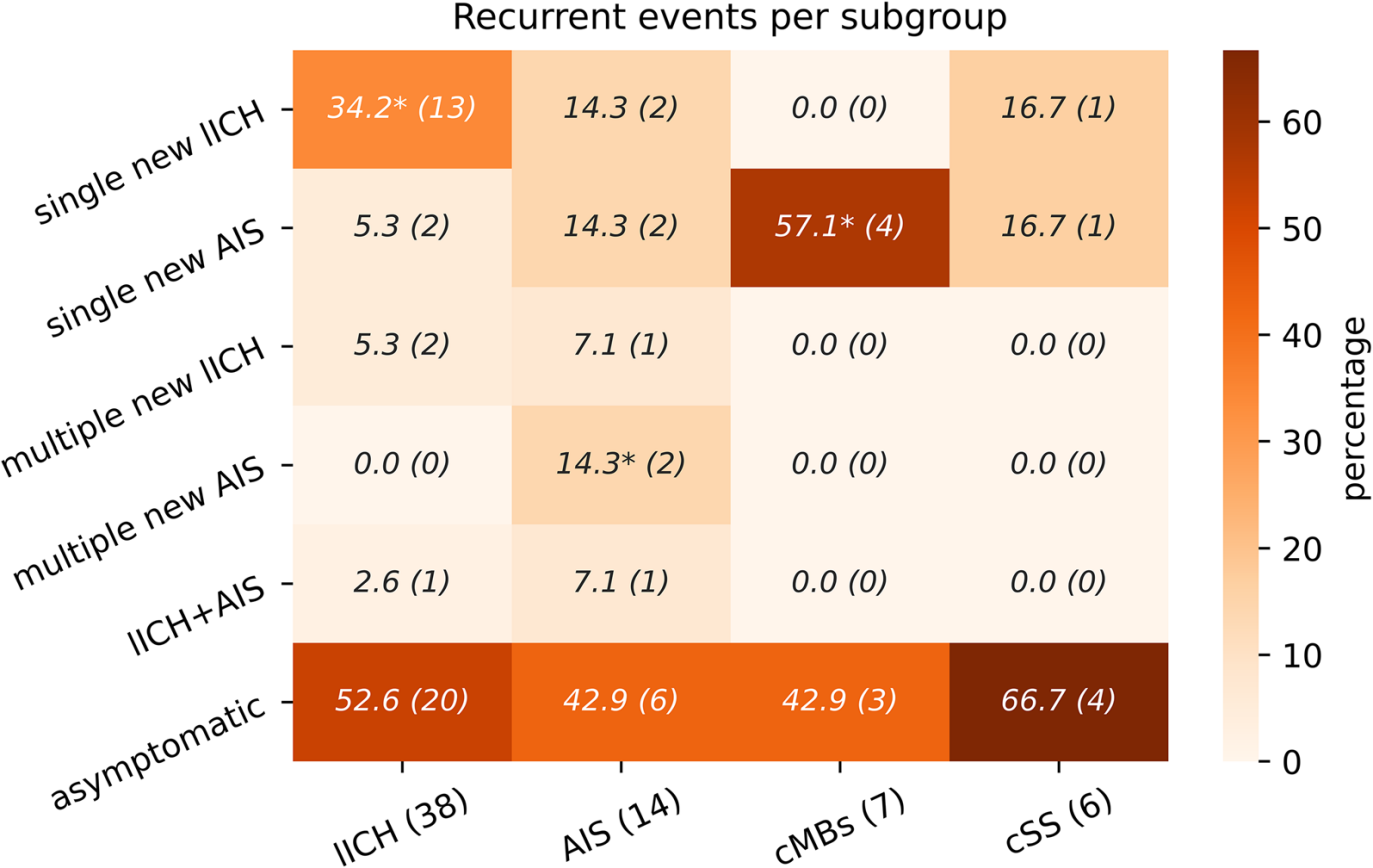
Recurrent events (on left hand side) grouped by index event (at bottom line); * = significant compared to the three other groups each (cross-row) (numbers: percentages per subgroup, number in parentheses: absolute patient numbers)

**Fig. 3:**
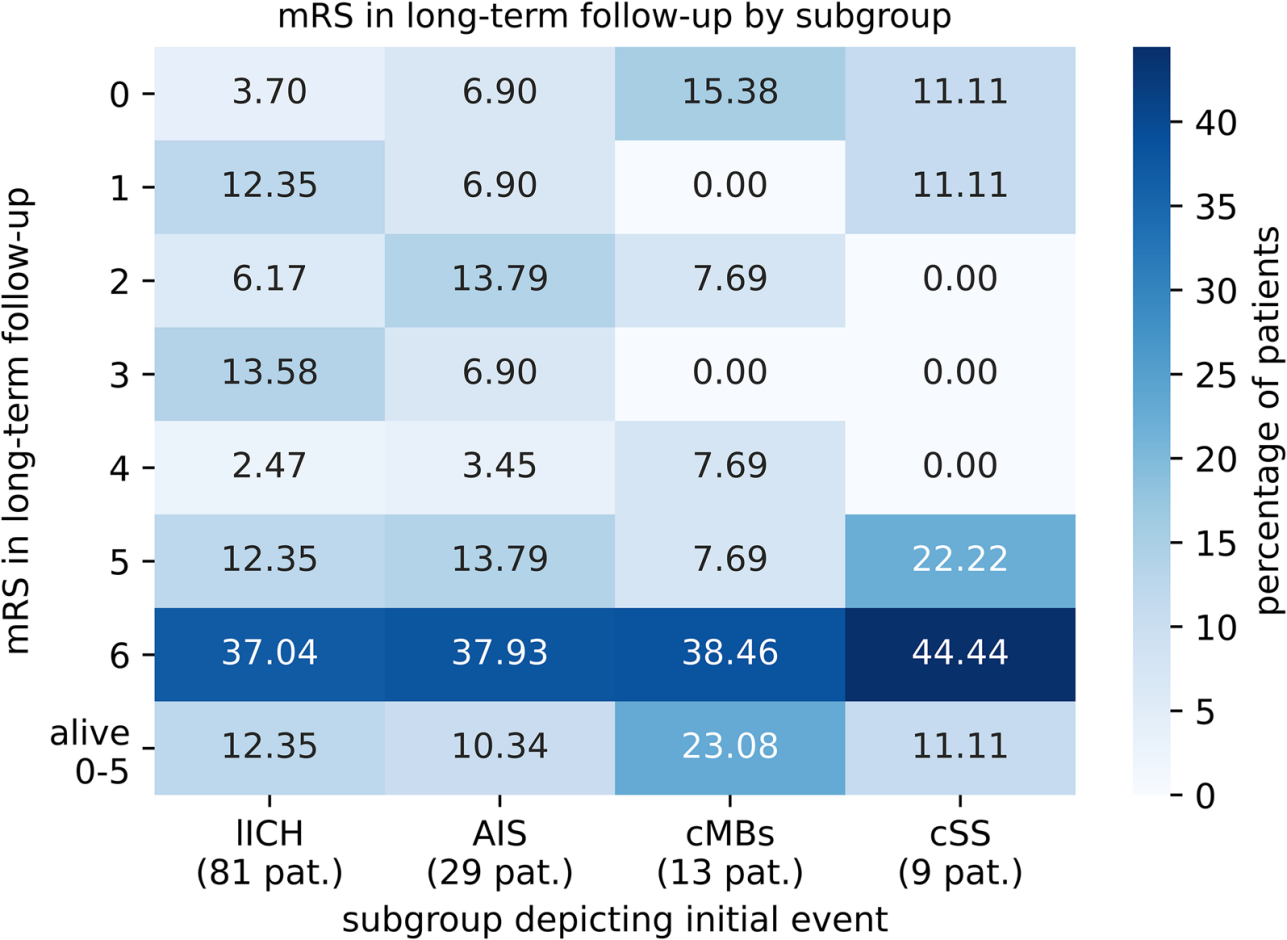
Heatmap of the different mRS scores upon long term follow up, split by subgroups (numbers given within the map: percentages; total number in the long-term follow-up: below the group classifications)

Furthermore, patients in the lICH-group had a significantly higher percentage of single new lICHs compared to the other three groups, whereas patients in AIS-group had a significantly higher percentage of multiple new AIS (each *p* < 0.00015). cMBs as index event predisposed more for AIS (*p* < 0.0016, Fig. 2).In the long-term mRS-analysis, the most frequent mRS was 6 (death). Other than that, no relevant differences could be seen between the different subgroups for mRS (*p*-value in chi square test > 0.84) (Fig. 3).

### MRI analysis

From all patients, 79.2% obtained at least one MRI with 28.8% obtaining more than one study.

**Fig. 4:**
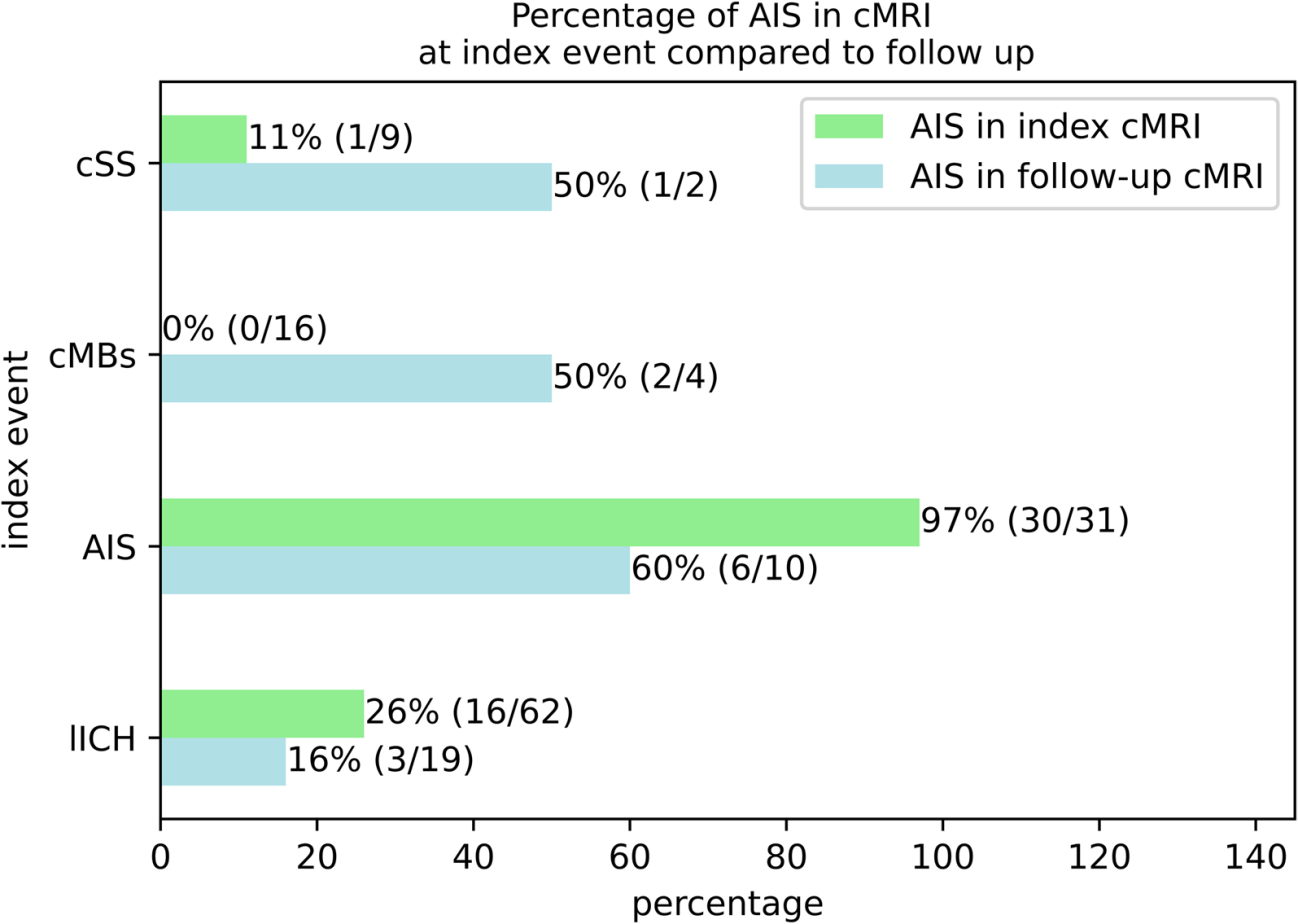
Percentages of AIS in cMRI at index event and in follow up, grouped by index event (numbers in parentheses: affected and overall patients)

Patients of the lICH-group had a trend towards lower probability of MRI lesions indicative of AIS in follow-up MRI than patients of the AIS-group, but overall chi-square testing on AIS in follow-up MRI did not reach statistical significance (*p* > 0.174). (Fig. 4). Acute stroke (clinically asymptomatic) was also diagnosed in non-AIS subgroups in the first MRI, especially in the lICH-group with 15 out of 56 patients affected. Subgroup analysis of the localization of the ischemic lesions in the AIS group showed the highest percentage for subcortical ischemic lesions (63.3%) followed by cortical ischemic lesions (20%) and mixed cortical/subcortical lesions (16.7%).

**Fig. 5:**
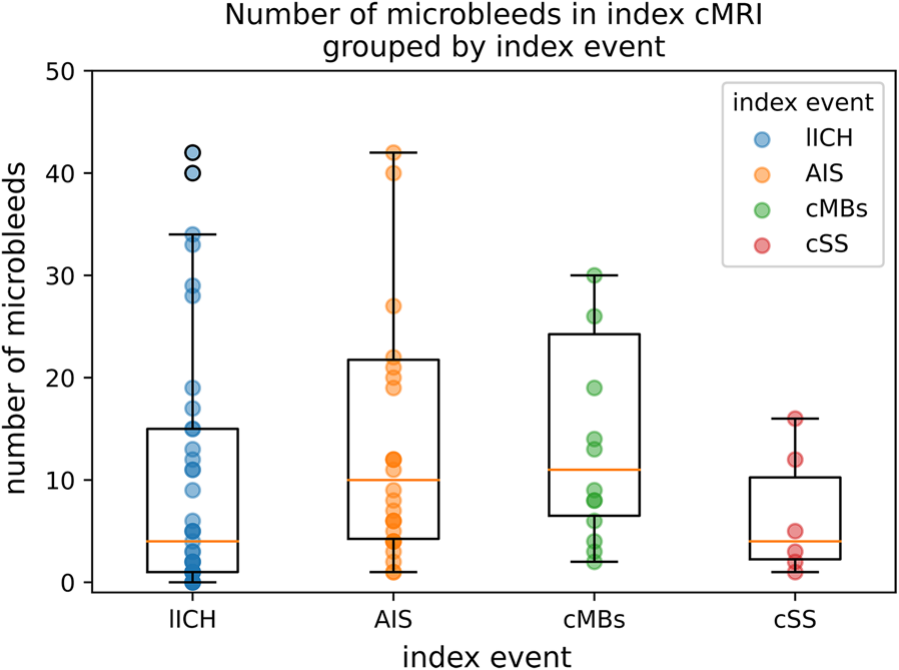
Number of microbleeds in index MRI grouped by index event for patients with the diagnosis of probable CAA. Each patient is represented by one data point in the plot, overlying are boxplots. Please note that a cut-off was set at 50 cMBs

The number of cMBs on first MRI was similar in the four subgroups and when considering patients with probable CAA diagnosis only (ANOVA: *p*-value > 0.20) (Fig. 5).

Interestingly, a trend towards fewer cMBs in lICH-group was seen when compared to cMBs- group—even when considering probable CAA patients only (*p* < 0.088 in t-test). The mean number of cMBs in the lICH-group was 10.7 with a median of 2 when considering all patients and a mean of 13.7 with a median of 4 when considering only patients with probable CAA. In the cMBs-group all patients had the diagnosis of a probable CAA with a mean of 38.4 and a median of 11 microbleeds.

There was no clear correlation between the age at index event and the number of cMBs with a trend to fewer cMBs in patients that are older at index event (Pearson’s r = − 0.13 (lICH), − 0.19 (AIS), − 0.61 (cMBs) and 0.57 (SAB/cSS)).

In patients with at least one follow-up MRI, an increase in the number of cMBs was seen in 66.7% of the lICH-group as compared to 90% of AIS-group. Only in the lICH- and AIS-group also a regress of cMBs was seen in 16.7% vs. 10%.

Overall, cSS was seen in 30.3% of index MRIs. In detail, it was seen in 75.0% of cSS/SAH-group, 40.0% of cMBs-group, 33.3% of AIS-group, and 19.6% of lICH-group. Two-tailed t-testing revealed a significant difference of cSS in lICH when compared to the three other subgroups (*p* < 0.022). Half of the patients of the cSS-group (50%) had a disseminated cSS, whereas in the AIS-group 80.0% of patients had a focal cSS.

The highest number of residual lesions in MRI at index event indicative of former ischemic or hemorrhagic strokes was found in the cSS-group (62.5%), followed by lICH-group (28.6%), AIS-group (26.7%) and cMBs-group (13.3%).

## Discussion

In this retrospective study, we demonstrate that the subsequent clinical course of CAA is significantly influenced by the patients’ initial clinical presentation.

cMBs as index event predisposed patients to recurrent single AIS. In addition, patients with cMBs may develop some type of protective mechanism shifting away essential functions from the regions prone to bleed, which may also prevent further bleedings due to ongoing neuroplasticity, similar to patients with hemodynamically relevant stenosis of a cerebral vessel who suffer an AIS in the same region of the brain and are in part protected by ongoing collateral vessel formation [17]. Former studies already showed the lower case fatality in patients with cMBs which then have a lICH [18]. Thus, cMBs in CAA may confer some sort of lICH-protection. However, our results are contrary to a former study, which showed cMBs in 69% of patients with spontaneous lICH and 40% of patients with ischemic cerebrovascular disease [19]. This is most likely explained by the fact that the authors included a mixed collective of elderly subjects with an overlap of classical cerebral microangiopathy, cMBs, and ischemic stroke. They showed that cerebral microangiopathy (lacunes, leukoaraiosis) is associated with the highest prevalence (57%) of cMBs among patients with ischemic stroke. CAA is a cerebral type II microangiopathy with overlap with type 1 by virtue of classical cerebrovascular risk factors especially hypertension. Yet, in our study all the patients in AIS subgroup fulfilled the modified Boston criteria for probable CAA. Thus, whether AIS is due to type 1 or 2 or both microangiopathies cannot be determined but remains an important finding stressing the need for optimal vascular risk factor treatment, especially hypertension.

The high number of recurrent TFNEs we could show was already described in a former study that showed recurrent TFNEs in 40% of patients with cSAH meeting mBC for probable CAA [20]. As these events are relevant for the clinical and socio-economical outcome (e.g. by losing a driving license, workplace restrictions, etc.), mainly rely on clinical reasoning and are less impressive than lICH or AIS, they must not be underdiagnosed.

Former studies showed that patients who present with cSS as the initial manifestation of CAA are at high risk for further events in general and have a poor outcome with a similarly high risk for future lICH and long-term mortality as CAA patients after primary lICH [21]. Moreover 62% of DWI lesions in CAA-patients were related to areas of cSS [22].

AIS as the primary manifestation of CAA is likely to be underdiagnosed since DWI lesions in CAA patients are often discussed as an epiphenomenon of CAA. Yet, a former study revealed that 130 DWI and T2* lesions examined histopathologically were in 100% acute microinfarcts [23].

CAA development and prognosis may be related to index vasculopathy. Particularly in the AIS-group, especially for the patients with subcortical strokes, an overlap with other microangiopathic vessel diseases may be discussed.

Secondary prevention in CAA patients with ischemic events is challenging and should be based on a stringent evaluation of all risk factors, especially for those with atrial fibrillation or other indications for anticoagulation. This is aggravated by the fact that almost one-fourth of CAA-patients with lICH suffer from coincident atrial fibrillation [24].

A major limitation of our study is the retrospective study design implying the risk of inclusion bias, especially that patients in lICH-group are more likely to be detected than patients of the other groups due to symptom severity and inclusion by cCT only.

## Conclusions

Cerebral amyloid angiopathy is a degenerative neurovascular disease with distinct phenotypes at the time of initial diagnosis. Our study, following up 149 patients over an average of 6.9 years, shows that the initial neurological manifestation defines the individual course of the disease, resulting in morbidity and specific recurrence risks. Prospective phenotypic CAA-registries may pave the way for specific secondary prophylaxis and are urgently needed to confirm our findings.

## Supplementary Information

Additional file 1. Boxplot of ages at index event split by gender. Each dot depicts one patient, total number 116. Overall, women were significantly older than men at the index event (p < 0.01, two-sided t-test, mean age (women) = 74.23 years, mean age (men) = 70.65. Orange lines show the median of each group (76 for females, 71 for males).

## Data Availability

The datasets used and/or analysed during the current study are available from the corresponding author on reasonable request.

## Abbreviations

CAA: Cerebral amyloid angiopathy
ICH: Intracerebral hemorrhage
lICH: Lobar ICH
cSAH: Cortical subarachnoidal hemorrhages
cSS: Cortical superficial siderosis
cMRI: Cerebral magnetic resonance imaging
cMB: Cerebral microbleeding
AIS: Acute ischemic stroke
TFNE: Transient focal neurological episodes
sMRS: Simplified modified Rankin Scale
PWML: Periventricular white matter lesion
DWL: Deep white matter lesions
mBC: Modified Boston criteria

## References

[1] Charidimou A, Gang Q, Werring DJ (2012). Sporadic cerebral amyloid angiopathy revisited: Recent insights into pathophysiology and clinical spectrum. Journal of Neurology, Neurosurgery and Psychiatry.

[2] Mehndiratta P, Manjila S, Ostergard T, Eisele S, Cohen ML, Sila C, Selman WR (2012). Cerebral amyloid angiopathy–associated intracerebral hemorrhage: Pathology and management. Neurosurgical Focus.

[3] Wardlaw JM, Smith C, Dichgans M (2013). Mechanisms of sporadic cerebral small vessel disease: Insights from neuroimaging. The Lancet Neurology.

[4] Yamada M (2015). Cerebral amyloid angiopathy: Emerging concepts. Journal of Stroke.

[5] Yeh SJ, Tang SC, Tsai LK, Jeng JS (2014). Pathogenetical subtypes of recurrent intracerebral hemorrhage: Designations by smash-u classification system. Stroke.

[6] Charidimou A, Boulouis G, Gurol ME, Ayata C, Bacskai BJ, Frosch MP, Viswanathan A, Greenberg SM (2017). Emerging concepts in sporadic cerebral amyloid angiopathy. Brain.

[7] Arima H, Tzourio C, Anderson C, Woodward M, Bousser MG, MacMahon S, Neal B, Chalmers J (2010). PROGRESS Collaborative Group: Effects of perindopril-based lowering of blood pressure on intracerebral hemorrhage related to amyloid angiopathy: The progress trial. Stroke.

[8] Hofmeijer J, Kappelle LJ, Klijn CJM (2015). Antithrombotic treatment and intracerebral haemorrhage: Between scylla and charybdis. Practical Neurology.

[9] Linn J, Halpin A, Demaerel P, Ruhland J, Giese AD, Dichgans M, Van Buchem MA, Bruckmann H, Greenberg SM (2010). Prevalence of superficial siderosis in patients with cerebral amyloid angiopathy. Neurology.

[10] Bruno A, Shah N, Lin C, Close B, Hess DC, Davis K, Baute V, Switzer JA, Waller JL, Nichols FT (2010). Improving modified rankin scale assessment with a simplified questionnaire. Stroke.

[11] Lees, K. (2014). *Sits open, how to perform modified rankin scale assessments: Training, questions and scoring* (pp. 1–15).

[12] Charidimou A, Linn J, Vernooij MW, Opherk C, Akoudad S, Baron JC, Greenberg SM, Jäger HR, Werring DJ (2015). Cortical superficial siderosis: Detection and clinical significance in cerebral amyloid angiopathy and related conditions. Brain.

[13] Gregoire SM, Chaudhary UJ, Brown MM, Yousry TA, Kallis C, Jäger HR, Werring DJ (2009). The microbleed anatomical rating scale (mars): Reliability of a tool to map brain microbleeds. Neurology.

[14] Zhang, H.-L., Linn, J., Bruckmann, H., & Greenberg, S. M. (2010). Prevalence of superficial siderosis in patients with cerebral amyloid angiopathy. *Neurology*, *75*(17), 1571; author reply 1571. 10.1212/WNL.0b013e3181f002c1 10.1212/WNL.0b013e3181f002c1 20975060

[15] Fazekas F, Chawluk JB, Alavi A, Hurtig HI, Zimmerman RA (1987). MR signal abnormalities at 1.5 T in alzheimer's dementia and normal aging. AJR.

[16] Wardlaw JM, Lewis SC, Keir SL, Dennis MS, Shenkin S (2006). Cerebral microbleeds are associated with lacunar stroke defined clinically and radiologically, independently of white matter lesions. Stroke.

[17] Lin M, Tsivgoulis G, Alexandrov AV, Chang JJ (2015). Factors affecting clinical outcome in large-vessel occlusive ischemic strokes. International Journal of Stroke.

[18] Samarasekera N, Fonville A, Lerpiniere C, Farrall AJ, Wardlaw JM, White PM, Smith C, Al-Shahi Salman R, Addison A, Ahmad K, Alhadad S (2015). Lothian Audit of the Treatment of Cerebral Haemorrhage Collaborators: Influence of intracerebral hemorrhage location on incidence, characteristics, and outcome: Population-based study. Stroke.

[19] Koennecke H (2006). Cerebral microbleeds on MRI: Prevalence, associations, and potential clinical implications. Neurology.

[20] Charidimou A, Peeters A, Fox Z, Gregoire SM, Vandermeeren Y, Laloux P, Jäger HR, Baron JC, Werring DJ (2012). Spectrum of transient focal neurological episodes in cerebral amyloid angiopathy multicentre magnetic resonance imaging cohort study and meta-analysis. Stroke.

[21] Wollenweber FA, Opherk C, Zedde M, Catak C, Malik R, Duering M, Konieczny MJ, Pascarella R, Samões R, Correia M, Martí-Fàbregas J (2019). Prognostic relevance of cortical superficial siderosis in cerebral amyloid angiopathy. Neurology.

[22] Beitzke M, Enzinger C, Pichler A, Wünsch G, Fazekas F (2018). Acute diffusion-weighted imaging lesions in cerebral amyloid angiopathy-related convexal subarachnoid hemorrhage. Journal of Cerebral Blood Flow & Metabolism.

[23] Ter Telgte A, Scherlek AA, Reijmer YD, van der Kouwe AJ, van Harten T, Duering M, Bacskai BJ, de Leeuw FE, Frosch MP, Greenberg SM, van Veluw SJ (2020). Histopathology of diffusion-weighted imaging-positive lesions in cerebral amyloid angiopathy. Acta Neuropathologica.

[24] Kaiser J, Schebesch KM, Brawanski A, Linker RA, Schlachetzki F, Wagner A (2019). Long-term follow-up of cerebral amyloid angiopathy-associated intracranial hemorrhage reveals a high prevalence of atrial fibrillation. Journal of Stroke and Cerebrovascular Diseases.

