# Supplementary material for: Index event of cerebral amyloid angiopathy (CAA) determines long-term prognosis and recurrent events (retrospective analysis and clinical follow-up)
Source: Neurol Res Pract. 2021 Sep 27;3:51. doi: 10.1186/s42466-021-00152-x (PMC8474746; doi:10.1186/s42466-021-00152-x)
Supplement: Supplementary file 1 — Boxplot of ages at index event split by gender. Each dot depicts one patient, total number 116. Overall, women were significantly older than men at the index event (p < 0.01, two-sided t-test, mean age (women) = 74.23 years, mean age (men) = 70.65. Orange lines show the median of each group (76 for females, 71 for males). [file 42466_2021_152_MOESM1_ESM.pdf]

Additional files:

Additional file 1:

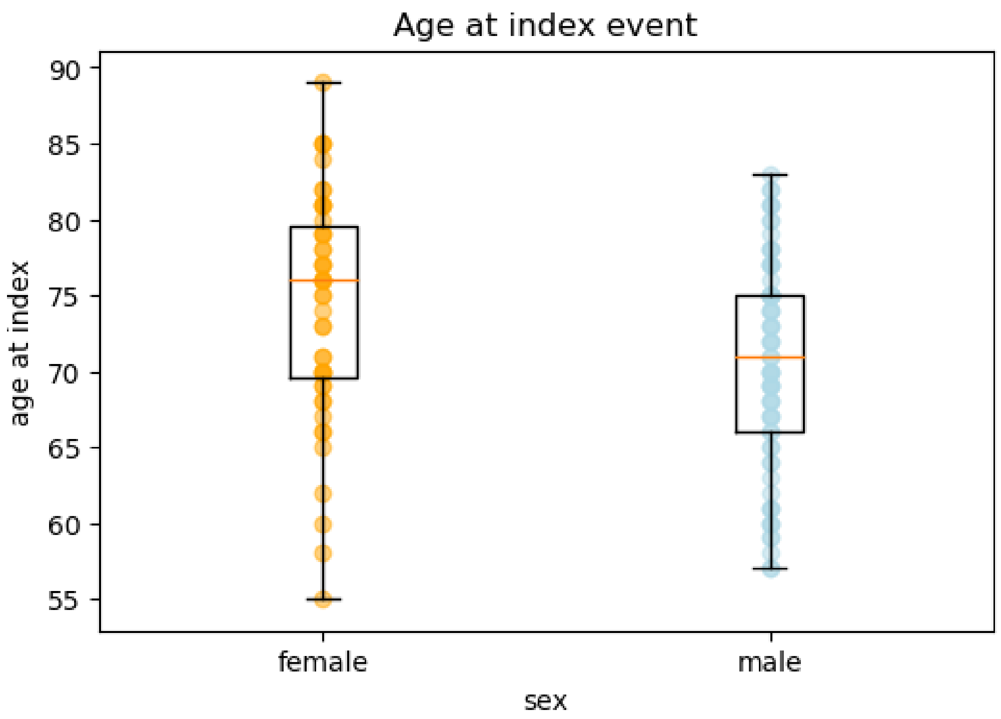

Appendix 2:

	Group 1			Group 2		
**at index event**	**in total (123)**	ICH (91)	AIS (32)	**in total (26)**	cMBs (16)	SAB/cSS (10)
percentage TFNE	1.6%(2)	2.2% (2)	0.0% (0)	19.2%(5)*	12.5% (2)	30.0% (3)
percentage no TFNE	81.3%(100)	78.0%(71)	90.6%(29)	53.8%(14)*	56.3%(9)	50.0%(5)
percentage epileptic seizures	17.1%(21)	19.8% (18)	9.4% (3)	26.9%(7)	31.3%(5)	20.0%(2)
**at long term follow up**	**in total (86)**	ICH (62)	AIS (24)	**in total (17)**	cMBs (10)	SAB/cSS (7)
percentage TFNE	2.3%(2)	1.6% (1)	4.2% (1)	17.6%(3)*	10.0% (1)	28.6% (2)
percentage no TFNE	62.8%(54)	56.5% (35)	79.2% (19)	29.4%(5)*	30.0% (3)	28.6% (2)
percentage epileptic seizures	34.9%(30)	41.9% (26)	16.7%(4)	52.9%(9)	60.0% (6)	42.9%(3)

Numbers to Figure 3. Group 2 showed statistically more TFNEs at the index event and less patients without TFNEs or epileptic seizures ($p < 1.3x10^{-3}$). A trend towards more epileptic seizures could be suspected but was far away from statistical significance due to small sample sizes. Furthermore, at long-term follow up more patients in group 2 had TFNEs and a less patients without TFNEs and/or epileptic seizures could be seen ($p < 6.7x10^{-3}$). Additionally, a trend towards more epileptic seizures in group two could be seen (no statistical significance). Hypothesis testing was done via binomial distribution with H0 being the percentages of events defined by group 1 tested against cases in group 2. Additionally, Fisher's exact test was performed. The given p-values depict the maximal p-values obtained by the two tests. Results were marked as significant (*) when $p<0.05$.
